# Transcriptome analyses provide insights into the homeostatic regulation of axillary buds in upland cotton (*G. hirsutum* L.)

**DOI:** 10.1186/s12870-020-02436-x

**Published:** 2020-05-24

**Authors:** Jianbin Shi, Ning Wang, Hong Zhou, Qinghua Xu, Gentu Yan

**Affiliations:** State Key Laboratory of Cotton Biology, Institute of Cotton Research of Chinese Academy of Agricultural Sciences, NO. 38, Huanghe Road, Anyang City, 455000 Henan Province China

**Keywords:** Upland cotton, Axillary bud, Transcriptome, DEGs

## Abstract

**Background:**

The axillary bud is an important index of cotton plant-type traits, and the molecular mechanism of axillary bud development in upland cotton has not yet been reported. We obtained a mutant (designated mZ571) with a high-budding phenotype in axillary bud development from the low-budding phenotype variety *G. hirsutum* Z571 (CCRI 9A02), which provided ideal materials for the study of complex regulatory networks of axillary bud development. In this study, RNA sequencing was carried out to detect gene expression levels during three stages of axillary buds in Z571 (LB, low budding) and mZ571 mutant (HB, high budding).

**Results:**

A total of 7162 DEGs were identified in the three groups (HB-E vs. LB-E, HB-G1 vs. LB-G1, HB-G2 vs. LB-G2), including 4014 downregulated and 3184 upregulated DEGs. Additionally, 221 DEGs were commonly identified in all three groups, accounting for approximately 3.09% of the total DEGs. These DEGs were identified, annotated and classified. A significant number of DEGs were related to hormone metabolism, hormone signal transduction, and starch and sucrose metabolism. In addition, 45, 22 and 9 DEGs involved in hormone metabolic pathways and 67, 22 and 19 DEGs involved in hormone signal transduction pathwayspathway were identified in HB-E vs. LB-E, HB-G1 vs. LB-G1, and HB-G2 vs. LB-G2, respectively, suggesting that endogenous hormones are the primary factors influencing cotton axillary bud growth. Hormone and soluble sugar content measurements revealed that mZ571 exhibited higher concentrations of zeatin, gibberellins and soluble sugar in all three stages, which confirmed that these hormone metabolism-, hormone signal transduction- and starch metabolism-related genes showed interaction effects contributing to the divergence of axillary bud growth between mZ571 and Z571.

**Conclusions:**

Our results confirmed the importance of endogenous hormones and sugars in the development of axillary buds, and we found that mZ571 plants, with a high-budding phenotype of axillary buds, exhibited higher endogenous hormone and sugar concentrations. Overall, we present a model for the emergence and development of cotton axillary buds that provides insights into the complexity and dynamic nature of the regulatory network during axillary bud emergence and development.

## Background

The axillary buds are an important agronomic trait that shapes plant structure, biomass and yield. In most higher plants, the branches originate from axillary meristems, while the main stem is derived from the apical meristems [1–3]. In different species, axillary buds show different degrees of development and growth during the vegetative period [4]. In the vegetative reproductive growth of *Arabidopsis thaliana*, the formation of axillary buds is inhibited until the start of flowering [4–6]. Similarly, in pea (*Pisum sativum*), the outgrowth of axillary buds is usually inhibited after the transformation of axillary meristems to buds with immature leaves at most nodes [7]. In rice (*Oryza sativa*), the growth of axillary buds is released to form tillers because of its characteristic growth habit [3], while in maize (*Zea mays*), another monocot species, the outgrowth of the axillary buds remains relatively restricted [8]. In sorghum (*Sorghum bicolor*), the *Nondormant Axillary Bud 1* gene was identified from a mutant plant and functionally characterized, and the mutant showed an increase in tillers and reduced plant height [1].

Plant hormones are the primary factors involved in the regulation of axillary bud growth. Three types of hormones, auxin, cytokinin and gibberellins (GAs), have long been studied. Among these hormones, auxin supplied by the terminal bud indirectly inhibits the growth of the axillary bud, while cytokinin directly induces the outgrowth of the axillary bud [9]. Studies on broad bean (*Vicia faba*) showed that removing the shoot tip of broad bean can stimulate the outgrowth of axillary buds, while this outgrowth could be largely suppressed by spraying auxin on the head of the plant residues [10]. However, since auxin is produced by young leaves and transported to the stem without entering the bud, it is believed that auxin has an indirect effect on bud growth [11–13]. Cytokinin has long been regarded as a bud growth-promoting factor [14, 15]. Recent studies have suggested that cytokinin regulates bud growth by controlling auxin transport [16]. The outgrowth of axillary buds can be promoted by the exogenous supply of an appropriate dose of cytokinin [17–19]. A study on cytokinin synthesis and signalling mutants of Arabidopsis indicated that cytokinin is related to the branches of intact plants but not to the branches of headless plants [20]. GAs are important endogenous hormones and play important roles in the overall growth of crops [21]. The promotion of axillary bud outgrowth by GAs has been reported in many plants, such as rose [22], sweet cherry [23] and *Jatropha curcas* [18].

Cotton provides excellent natural fibre and is one of the most important economic crops in the world. In cotton production, simplified cultivation and mechanical harvesting are emerging trends that depend on whether the cotton plant type meets production requirements. Cotton varieties with ideal plant types not only accelerate mechanized cotton production but also exhibit better ventilation, light permeability and leaf falling effects. The overgrowth of axillary buds in cotton increases the workload of artificial pruning and is not conducive to mechanized management and harvesting. In addition, the ideal cotton plant type shows good ventilation and light transmittance and is not vulnerable to pests, thus reducing dependence on pesticides. In cotton plants with few axillary buds, it is less likely that fallen leaves will hang on the plants, thereby reducing impurities during the cotton harvest, which is conducive to improving the quality of fibre. Therefore, branch types with axillary buds are an important index of plant type traits that have been well studied in many different species [3, 5, 24–28]. The mechanisms of axillary bud growth and development have not been reported in cotton.

In this study, the upland cotton variety Z571 (CCRI 9A02) and an axillary bud mutant thereof (mZ571) were selected as materials for identifying the regulatory networks involved in the development of axillary buds in upland cotton. Z571 has an ideal plant type for simplified cultivation and machine mining because of its fewer axillary buds and vegetative shoot characteristics. The low-budding phenotype of the axillary buds in Z571 is helpful for the mechanization of cotton harvesting. mZ571 is a phenotypic mutant inbred line with a high-budding phenotype in the development of axillary buds compared with Z571. Z571 and mZ571 provide ideal materials with the same genetic background subject to little interference from the environment for the study of complex regulatory networks of axillary bud development. Similarly, the contents of three kinds of phytohormones (cytokinin, gibberellins and auxin) associated with axillary buds were detected to analyse the underlying regulatory mechanism in upland cotton. Additionally, transcriptome sequencing of the first stalk nodes of the two plant types was performed at four, five and six weeks to study the regulatory network in the early stage of axillary bud development. This research not only provides data for studying the molecular mechanism of cotton axillary bud development but also lays a solid foundation for ideal plant type breeding in cotton.

## Results

### Phenotype, endogenous hormones and soluble sugar content of *G. hirsutum* Z571 and its mutant mZ571

**Fig. 1:**
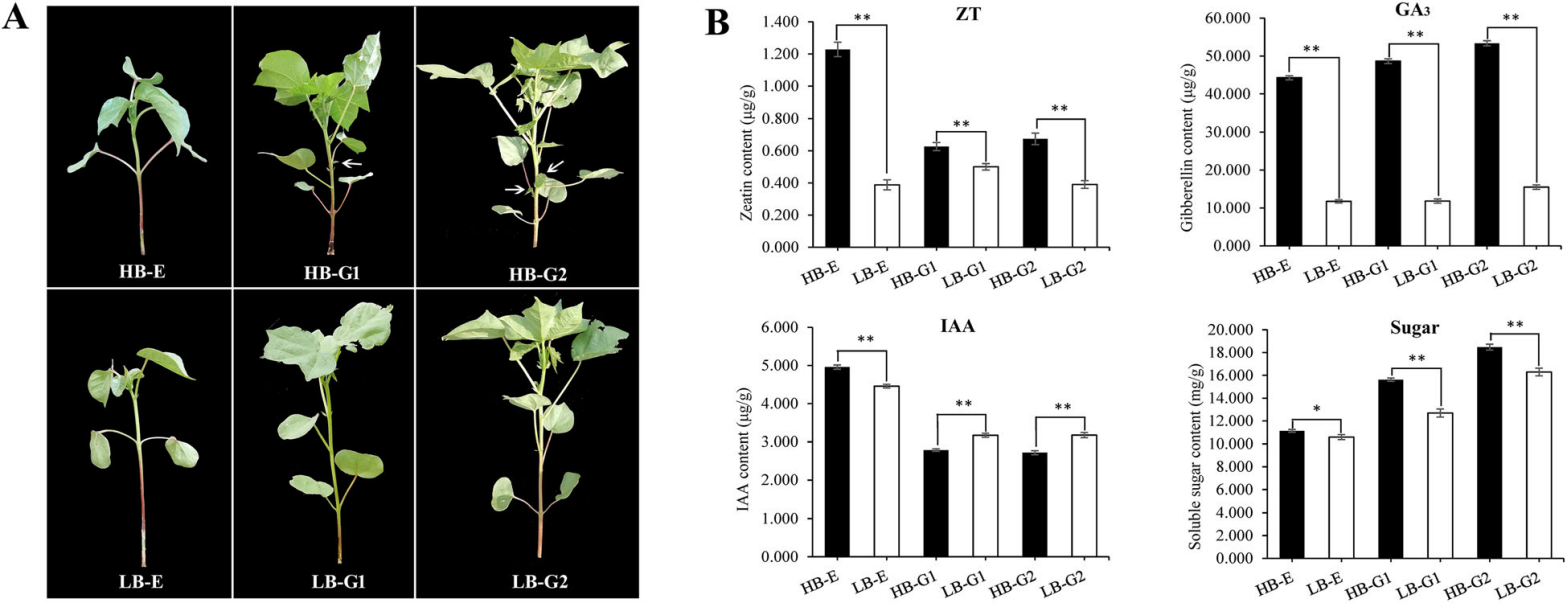
Phenotypic analysis and identification of DEGs in axillary buds at different stages in *G. hirsutum*. **a** The phenotypes of Z571 and mZ571 in three stages. **b** The changes in ZT, GA, IAA and soluble sugar concentrations at different stages of the two axillary bud types. “HB-E” and “LB-G” represent the “high-budding phenotype, early stage” and “low-budding, growth stage”, respectively. “*” and “**” indicate significant differences at *P* < 0.05 and *P* < 0.01, by the LSD test

The stalk nodes used in the present study were collected from three stages of axillary bud development at the first branch position. The development of axillary buds in Z571 is limited, while that in mZ571 is very rapid (Fig. 1a). To monitor changes in cytokinin, gibberellin and auxin during axillary bud development, we measured the concentrations of zeatin (ZT), gibberellins (Gas) and indole acetic acid (IAA) in six samples by HPLC [29]. ZT showed the highest concentration in HB-E and slightly decreased concentrations in HB-G1 and HB-G2, and the concentrations of ZT in LB-E and LB-G2 were significantly lower than those in HB-E and HB-G2. Both types of plant materials showed an increasing tendency of the GA concentration during all stages. In contrast, IAA showed a decreasing pattern during the first two stages and then remained stable in the third stage. However, the IAA concentrations of HB-G1 and HB-G2 were significantly lower than those of LB-G1 and LB-G2, respectively. In addition, with the development of the cotton plants, the soluble sugar content in the axillary buds of the two plant types gradually increased, and the soluble sugar contents of HB-E, HB-G1 and HB-G2 were higher than those of LB-E, LBL-G1 and LB-G2, respectively (Fig. 1b). These results suggested that endogenous hormones and sugars are critical regulators during cotton axillary bud development.

### RNA sequencing and transcriptome analysis

In total, 845,061,558 raw reads were generated from the HB-E, HB-G1, HB-G2, LB-E, LB-G1 and LB-G2 libraries (three replicates for each sample). The percentage of nucleotides with a quality score above 20 (Q20) was approximately 97.60% (Table S2). After the filtering of reads with adapters at the 3′ ends and quality scores below Q20, over 93.30% of the reads were confirmed as clean reads. As a result, 93.98–95.46% of the total clean reads were mapped to the upland cotton genome by using the TopHat2 upgrade version HISAT2 [30]. Multiple mapped reads and uniquely mapped reads accounted for 9.66–10.94% and 89.14–90.34% of these reads, respectively (Table S3). The mapped reads were assembled using StringTie software based on the reference genome, and compared with known transcripts, a total of 74,324 unigenes were generated.

### Functional annotation of unigenes

The functional annotation of the unigenes was carried out by comparison with five different databases. In total, 10,197 unigenes (approximately 13.72%) were successfully matched in all five databases (Table 1). These unigenes were categorized into 26 descriptions according to the eggNOG database functional classification among which unigenes involved in signal transduction mechanisms and transcription were highly represented (Fig. S1).

**Table 1:** Annotation of unigenes in five different databases

Database	Number	Percentage
Annotation in NR	70,889	95.38
Annotation in GO	46,000	61.89
Annotation in KEGG	11,968	16.1
Annotation in eggNOG	66,635	89.66
Annotation in Swissprot	55,926	75.25
In all database	10,197	13.72
Total unigenes	74,324	100.00

### Identification and analysis of DEGs

**Fig. 2:**
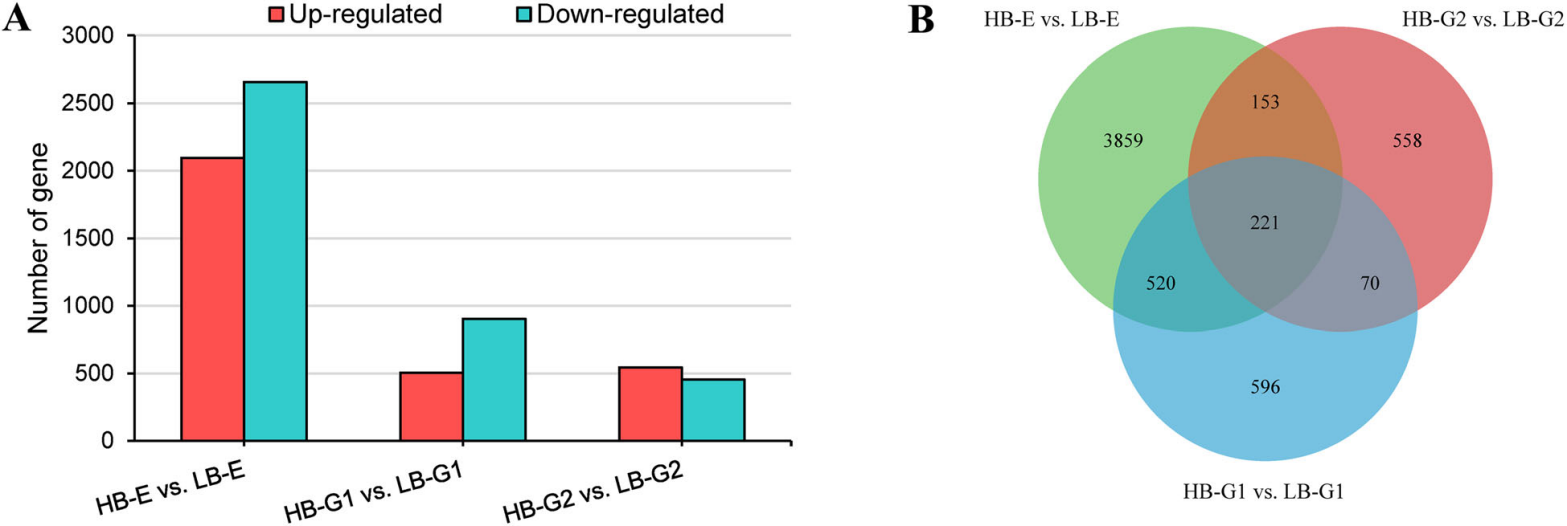
The identification of DEGs. **a** Numbers of DEGs in HB-E vs. LB-E, HB-G1 vs. LB-G1 and HB-G2 vs. LB-G2. **b** Venn diagram of the DEGs in the three groups. “HB-E” and “LB-G” represent the “high-budding phenotype, early stage” and “low-budding, growth stage”, respectively

FPKM values were used to represent the abundance of each gene. A cutoff of a log2 (fold change) > 1 and a *p*-value < 0.05 were used to define DEGs. As a result, 7162 unigenes were found to show significantly differential expression in all three groups, accounting for 9.64% of the total unigenes. Among the DEGs, 4014 were downregulated and 3148 were upregulated (Fig. 2a). Additionally, 221 DEGs were commonly identified in the two axillary bud types in the three comparison groups (HB-E vs. LB-E, HB-G1 vs. LB-G1, HB-G2 vs. LB-G2), representing approximately 3.09% of the total DEGs. (Fig. 2b).

### GO functional classification of DEGs

**Fig. 3:**
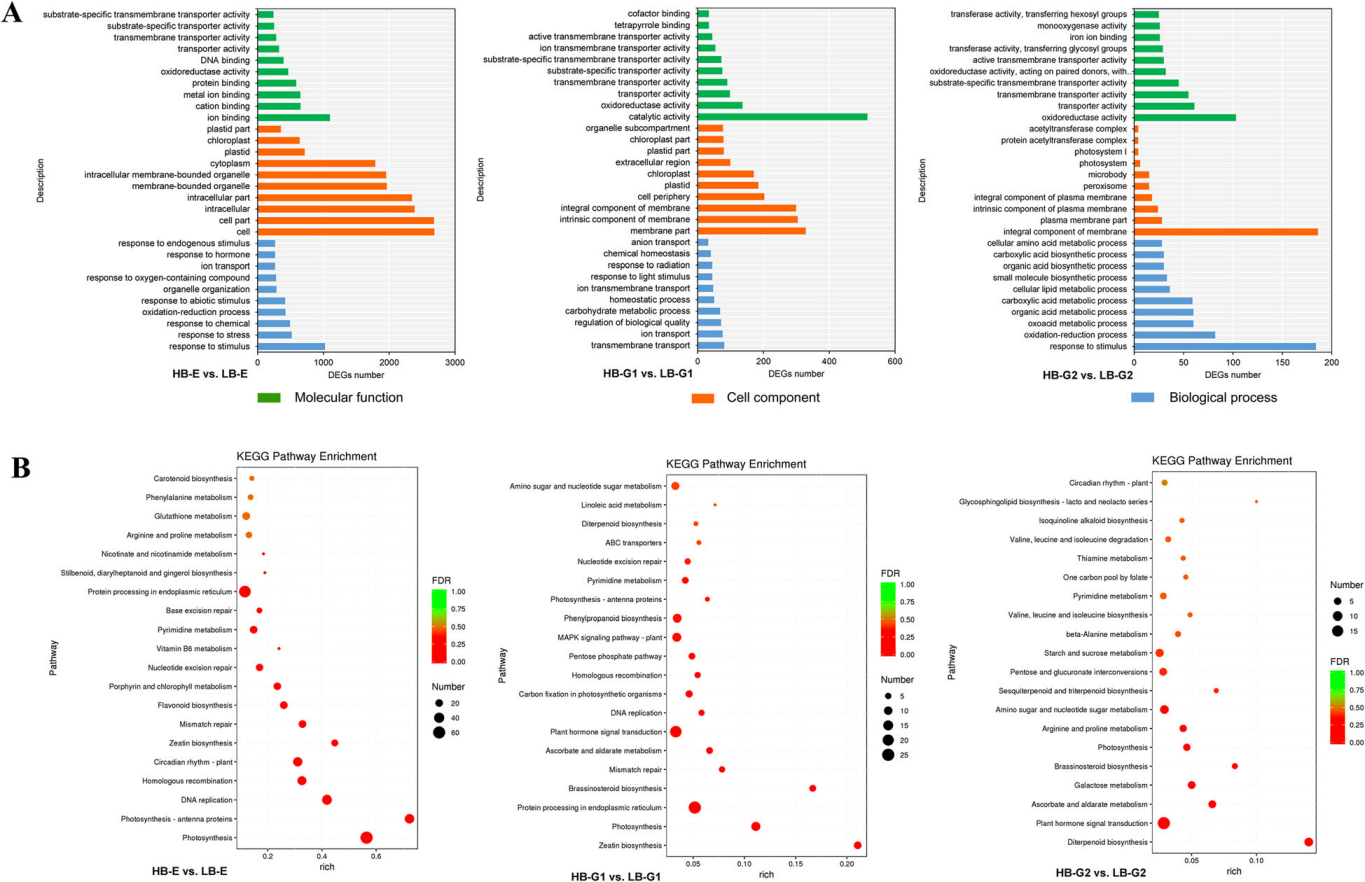
GO function and KEGG pathway classification of DEGs. **a** GO functional classification and numbers of DEGs in three groups. **b** KEGG pathways and numbers of DEGs in the three groups

Based on GO categories, the functions of all DEGs were assessed. The results showed that 3783 (79.59%), 1068 (75.91%) and 738 (73.65%) DEGs in HB-E vs. LB-E, HB-G1 vs. LB-G1 and HB-G2 vs. LB-G2, respectively were enriched in the three main categories of cellular components (CC), biological processes (BP) and molecular functions (MF) under the criterion of a *p*-value< 0.05 (Fig. 3a). The DEGs annotated in the CC category were related to cell and membrane parts. Under the BP category, the DEGs were involved in the response hormones and stimuli. The DEGs annotated in the MF category were mainly involved in ion binding, catalytic activity and oxidoreductase activity. In addition, 45 DEGs (7 upregulated, 38 downregulated) identified in HB-E vs. LB-E, 22 DEGs (3 upregulated, 19 downregulated) in HB-G1 vs. LB-G1, and 9 DEGs (5 upregulated, 4 downregulated) in HB-G2 vs. LB-G2 were involved in hormone metabolic processes, which indicates that endogenous hormones play a significant role in the development of axillary buds.

### Pathway analysis of DEGs based on KEGG

In total, 6893 DEGs were assigned to 123 pathways based on KEGG annotation, and 57 pathways were found to be significantly enriched under the criterion of *p*<0.05. The top 30 pathways with the most abundant DEGs involved in axillary bud development are shown in Fig. 3b, which included the photosynthesis, DNA replication, plant hormone signal transduction, photosynthesis-antenna proteins, and protein processing in the endoplasmic reticulum pathways. Among these pathways, plant hormone-associated, photosynthesis-associated, DNA replication and starch and sucrose metabolism pathways accounted for most of the DEGs. The top five pathways with the most abundant DEGs are shown in Table 2.

**Table 2:** Pathways of differentially expressed unigenes annotated in KEGG

NO.	Pathway annotated in KEGG	DEG number	Total number	P-value	Pathway ID	Level 1	Level 2
**HB-E vs. LB-E**							
1	Plant hormone signal transduction	67	666	0.138944617	ko04075	Environmental Information Processing	Signal transduction
2	Photosynthesis	61	108	2.63445E-36	ko00195	Metabolism	Energy metabolism
3	Protein processing in endoplasmic reticulum	56	485	0.022169926	ko04141	Genetic Information Processing	Folding, sorting and degradation
4	DNA replication	36	86	1.97547E-16	ko03030	Genetic Information Processing	Replication and repair
5	Photosynthesis - antenna proteins	34	47	4.46141E-26	ko00196	Metabolism	Energy metabolism
**HB-G1 vs. LB-G1**							
1	Protein processing in endoplasmic reticulum	25	485	2.21325E-05	ko04141	Genetic Information Processing	Folding, sorting and degradation
2	Plant hormone signal transduction	22	666	0.018159619	ko04075	Environmental Information Processing	Signal transduction
3	Photosynthesis	12	108	2.04012E-06	ko00195	Metabolism	Energy metabolism
4	MAPK signaling pathway - plant	12	353	0.058286698	ko04016	Environmental Information Processing	Signal transduction
5	Phenylpropanoid biosynthesis	11	320	0.063782317	ko00940	Metabolism	Biosynthesis of other secondary metabolites
**HB-G2 vs. LB-G2**							
1	Plant hormone signal transduction	19	666	0.000772591	ko04075	Environmental Information Processing	Signal transduction
2	Diterpenoid biosynthesis	8	57	5.31481E-07	ko00904	Metabolism	Metabolism of terpenoids and polyketides
3	Amino sugar and nucleotide sugar metabolism	8	276	0.023594718	ko00520	Metabolism	Carbohydrate metabolism
4	Starch and sucrose metabolism	7	277	0.060857028	ko00500	Metabolism	Carbohydrate metabolism
5	Ascorbate and aldarate metabolism	6	91	0.001004811	ko00053	Metabolism	Carbohydrate metabolism

“HB-E” and “LB-G” for “high-budding phenotype, early stage” and “low-budding, growth stage”, respectively

### qRT-PCR validation

**Fig. 4:**
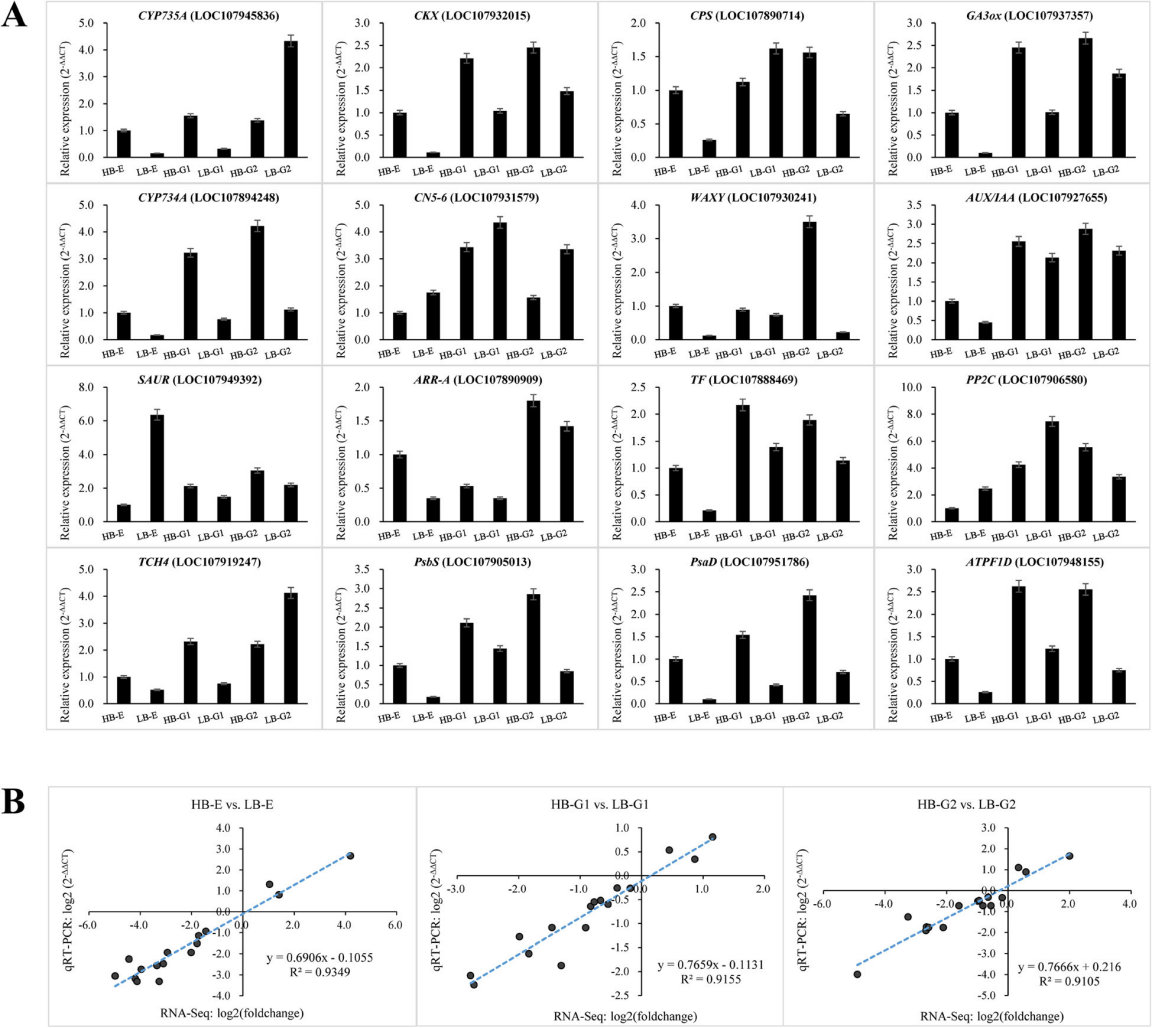
Validation of RNA-Seq data by qRT-PCR analysis. **a** Expression of representative genes in the different developmental stages of axillary buds validated by qRT-PCR. **b** Correlations of the fold changes of sixteen differentially expressed genes determined by RNA-Seq and qRT-PCR analysis

To verify the reliability of the RNA-Seq DEG data, sixteen DEGs (three photosynthesis-related genes, two zeatin biosynthesis genes, two starch and sucrose metabolism genes, two gibberellin biosynthesis genes, one brassinosteroid biosynthesis gene and six plant hormone signal transduction genes) were used for qRT-PCR analysis. The results showed that the expression patterns of these DEGs were mainly in agreement with the RNA-Seq results (Fig. 4a), with correlation coefficients (R^2^) > 0.91 (Fig. 4b). The results therefore confirmed the reliability of the RNA-Seq data.

### Analysis of DEGs involved in hormone biosynthesis and signal transduction

Previous studies have shown that endogenous hormones are crucial to plant growth and development. Here, several genes involved in the plant hormone signal transduction, zeatin, and gibberellin pathways were found to be differentially expressed between HB-E and LB-E, HB-G1 and LB-G1, and HB-G2 and LB-G2. For example, 14 and 10 DEGs were differentially expressed in the pathways of cytokinin (zeatin) and GA biosynthesis, respectively, which are widely believed to be involved in regulating cotton plant growth [31, 32].

**Fig. 5:**
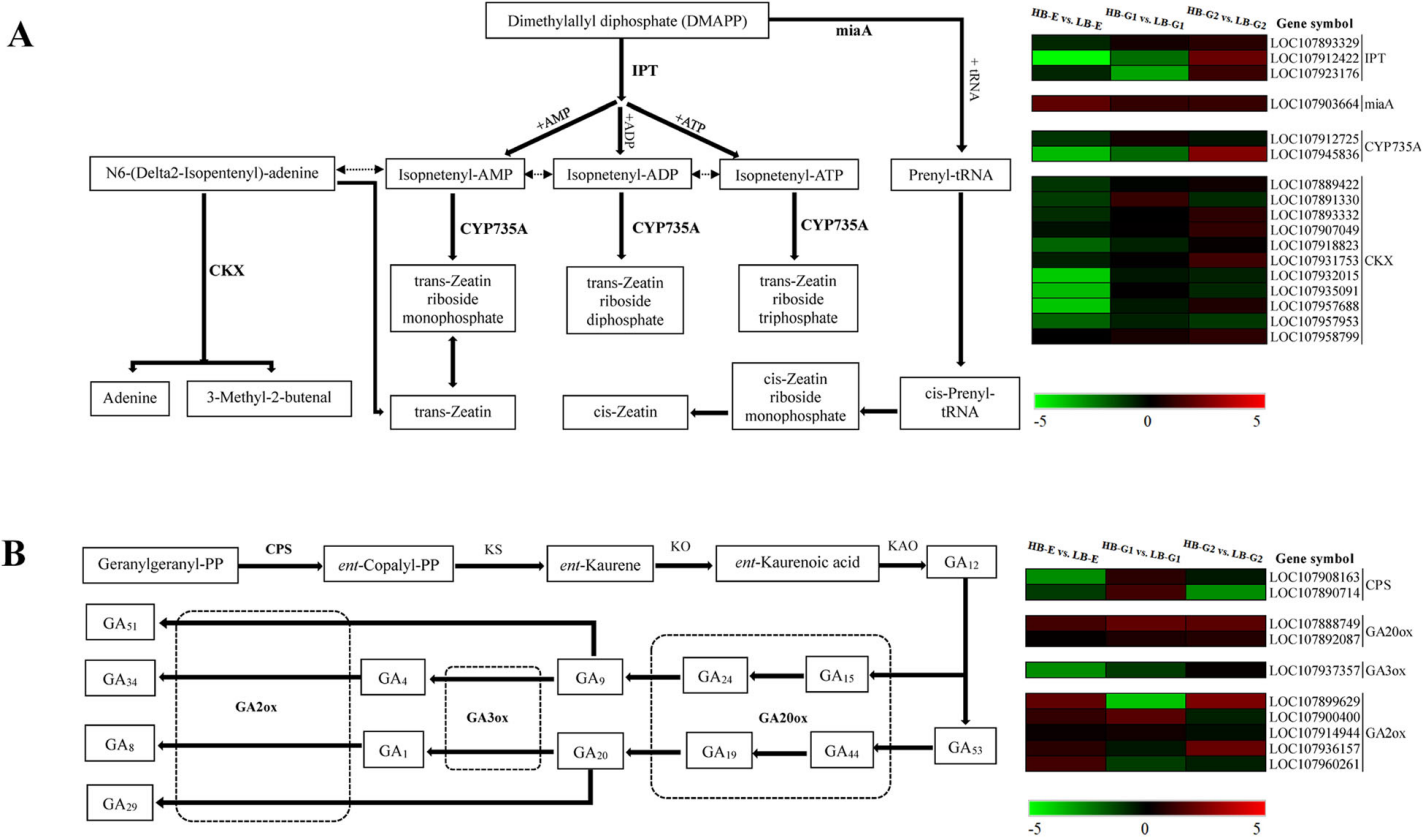
Transcript profiling of genes in the HB-E vs. LB-E, HB-G1 vs. LB-G1 and HB-G2 vs. LB-G2 groups. **a** The zeatin biosynthetic pathway. **b** The gibberellin biosynthetic pathway. “HB-E” and “LB-G” represent the “high-budding phenotype, early stage” and “low-budding, growth stage”, respectively. The colour scale from green to red represents relative expression levels in the heatmap

**Fig. 6:**
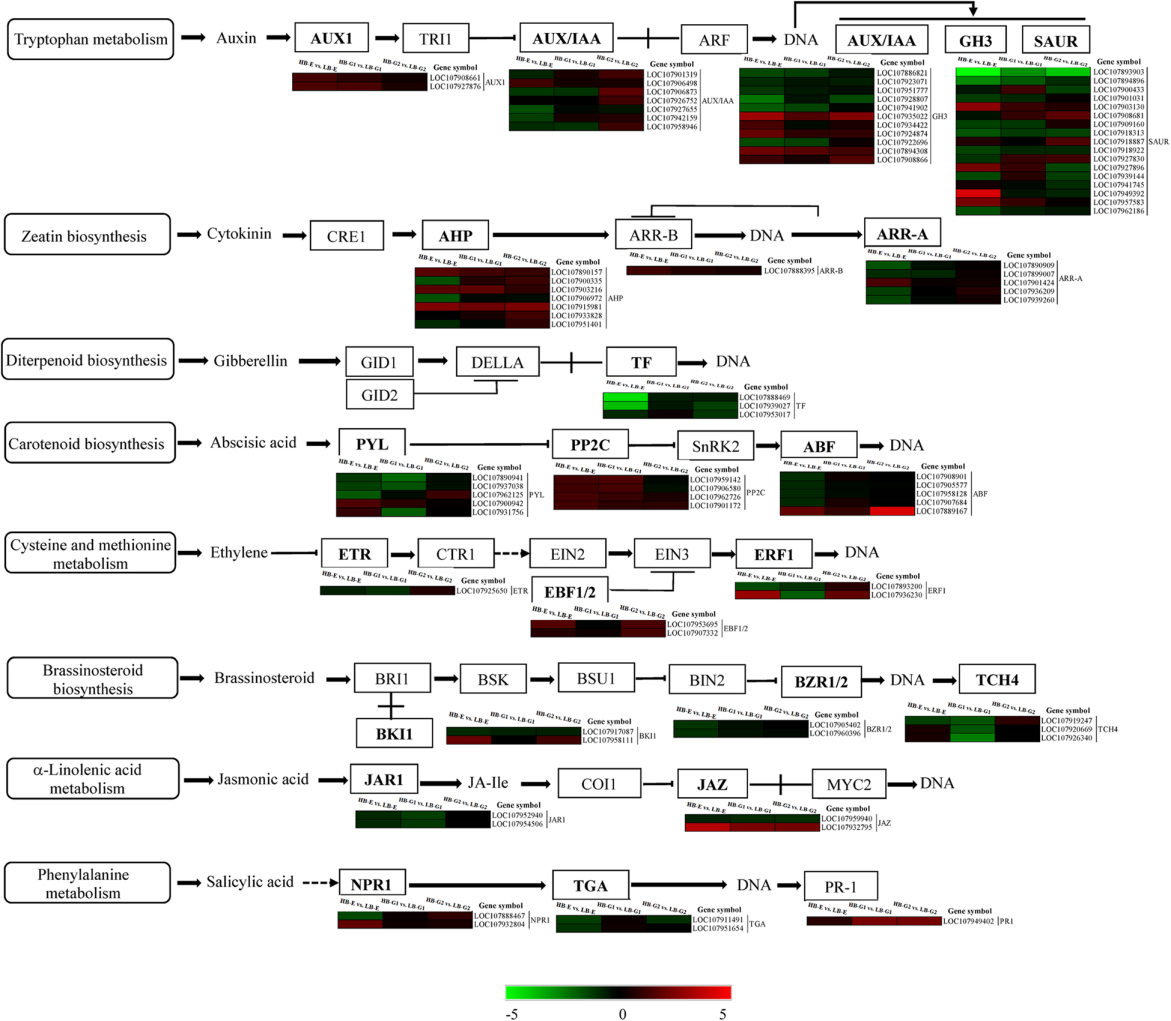
The plant hormone signal transduction pathways. “HB-E” and “LB-G” represent the “high-budding phenotype, early stage” and “low-budding, growth stage”, respectively. The colour scale from green to red represents the relative expression levels in the heatmap

In the zeatin biosynthesis pathway, the expression level of *miaA* was upregulated in all three stages of mZ571. One *IPT* (LOC107912422) and one *CYP735A* (LOC107945836) were upregulated, but most *CKX* genes were still downregulated in HB-G2 (Fig. 5a). The expression level of the first gene encoding carbumoyl pyrophosphate synthetase that enters the GA synthesis pathway, *CPS*, was slightly downregulated in HB-E and HB-G2, while there was no significant change in the HB-G1 stage. One *GA20ox* gene (LOC107888749) was significantly upregulated in HB-G1 and HB-G2. Three *GA2ox* genes (LOC107899629, LOC107936157, LOC107960261) were significantly downregulated in HB-G1. In addition, *GA3ox* (LOC107937357) was downregulated in HB-E, HB-G1 and HB-G2, but the rate of reduction gradually decreased (Fig. 5b). For *AUX1*, which is involved in auxin signal transduction, the expression level was significantly upregulated in HB-E and HB-G1 cells. One *AUX/IAA* (LOC107906498), four *CH3* (LOC107935022, LOC107934422, LOC107924874, LOC107894308), and four *SAUR* (LOC107903130, LOC107927896, LOC107949392, LOC107957583) genes were significantly upregulated in HB-E. Three *AUX/IAA* (LOC107901319, LOC107906873, LOC107926752), three *CH3* (LOC107935022, LOC107894308, LOC107908866) and two *SAUR* (LOC107908681, LOC107918887) genes were significantly upregulated in HB-G2 (Fig. 6). For *AHP*, *ARR-B* and *ARR-A*, genes involved in cytokinin (zeatin) signal transduction, two *AHP* (LOC107890157, LOC107903216) genes in HB-G1, one *ARR-B* (LOC107888395) and one *ARR-A* (LOC107901424) gene in HB-E, and one *AHP* (LOC107915981) gene in all three stages of mZ571 were significantly upregulated. The *BZR1/2* genes. Genes involved in brassinosteroid signal transduction, were downregulated in HB-E, after which their expression increased gradually in HB-G1 and HB-G2 but was still lower than that of LB. One *BKI1* (LOC107958111) gene was significantly upregulated in HB-E and HB-G2. There was no significant upregulation of *TCH4* genes. In addition, other genes involved in signal transduction, such as *PYL*, *PP2C*, and *ABF*, associated with abscisic acid; *ETR*, *EBF1/2*, and *ERF1*, associated with ethylene; *JAR1* and *JAZ*, associated with jasmonic acid; and *NPR1*, *TGA*, and *PR1*, associated with salicylic acid, also showed differential expression. One *ABF* (LOC107889167), one ERF1 (LOC107936230) and one JAZ (LOC107932795) gene were significantly upregulated in HB-E (Fig. 6).

### Analysis of DEGs involved in starch and sucrose metabolism

**Fig. 7:**
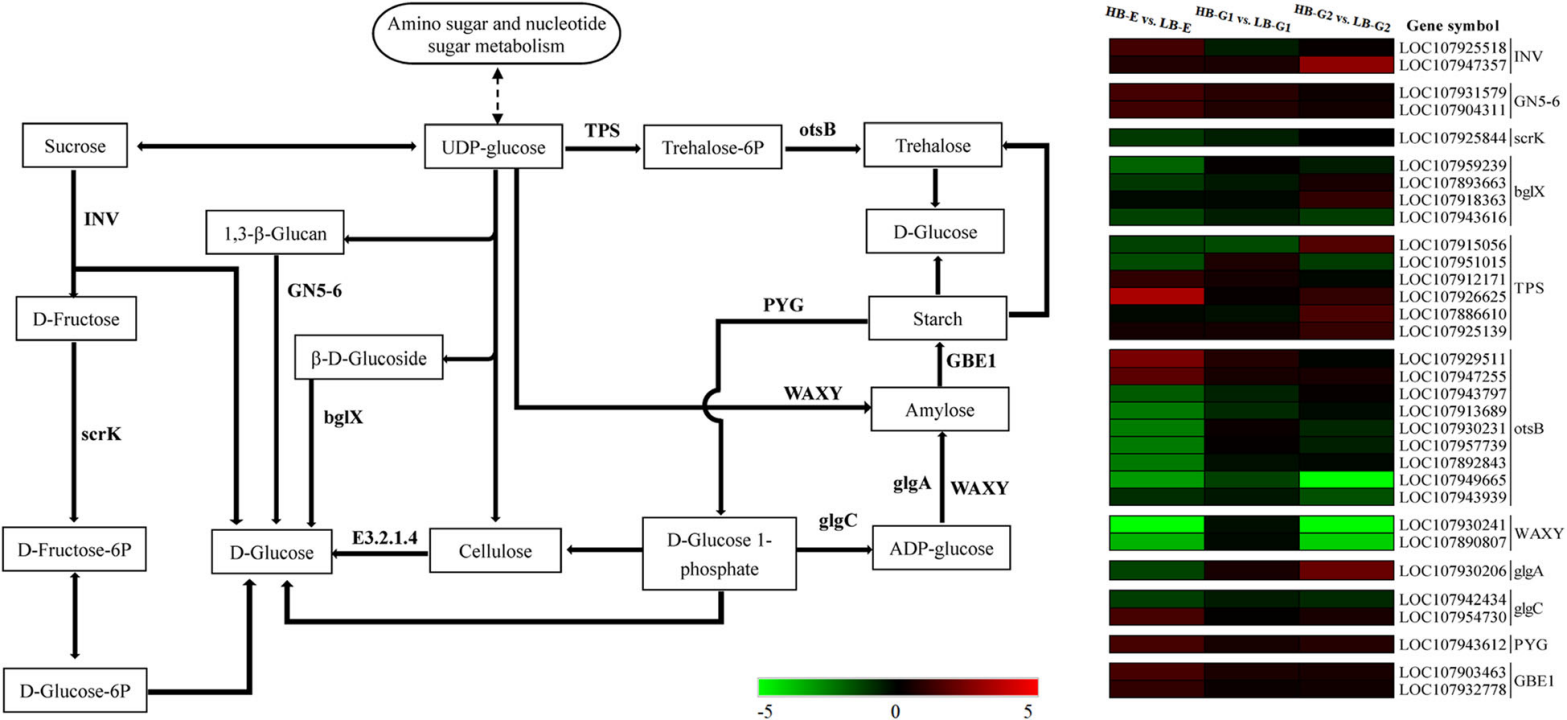
The starch and sucrose metabolism pathway. “HB-E” and “LB-G” represent the “high-budding phenotype, early stage” and “low-budding, growth stage”, respectively. The colour scale from green to red represents the relative expression levels in the heatmap

Sugar plays an important role in the growth and development of plants. Recent studies have shown that sugar is specifically an important signalling regulator of bud growth [33]. In the starch and sucrose metabolism pathway, *INV* was upregulated in HB-E and HB-G2 compared to LB-E and LB-G2. The expression levels of *GN5–6*, *PYG*, and *GBE1* were upregulated in HB-E, HB-G1 and HB-G2 cells. Among these genes, *GN5–6* presented the highest expression level in HB-E, which then gradually decreased in the HB-G1 and HB-G2 stages. The high expression of *GN5–6* guarantees the transformation of 1,3-β-glucan to D-glucose. *scrK*, *bglX*, and most *TPS* and *otsB* genes were downregulated in HB-E and HB-G1. One *TPS* (LOC107926625) and two *otsB* (LOC107929511, LOC107947255) genes were upregulated in all three stages of HB.HB In addition, *WAXY* exhibited a higher expression level in HB-E, then decreased in HB-G1 and significantly increased in HB-G2 (Fig. 7).

## Discussion

The plant type of a crop is the comprehensive expression of the morphological structure and unique physiological and ecological functions of a plant and is closely related to crop production and breeding; a favourable plant type can improve the leaf area coefficient, photosynthetic efficiency and fertility tolerance, thus increasing the harvest index [34–36]. The characteristics that affect the formation of different upland cotton plant types include the fruit branch type, number of shoot branches, number of fruiting branches, and plant height. Among these characteristics, the shoot branch, which is formed from the axillary bud, is one of the main factors affecting mechanical harvesting and the establishment of cultivation measures. Transcriptome research can provide new insight into the gene expression network of axillary bud development [37, 38].

### Cytokinin and gibberellin are important regulators of axillary bud development

In-depth research on the hormonal control of axillary buds has been performed over time. A series of branching mutants in pea, morning glory, rice, and Arabidopsis plants have been analyzed to further demonstrate that the activation of buds can be controlled by auxin transporter-based systems [27, 39–43]. Cytokines have the function of positively regulating meristems and are necessary to maintain them. In general, endogenous cytokinins are transported acropetally in the xylem sap to promote axillary bud outgrowth [14]. Gibberellin belongs to the diterpene family and is essential for the growth and development of plants [18]. Previous studies have also shown that a series of changes in endogenous cytokinin and auxin levels are key factors regulating axillary bud development [14, 44–46].

In this study, to elucidate the differential regulatory networks of genes involved in axillary bud emergence and development in upland cotton, a transcriptome analysis was performed in Z571 and its mZ571 mutant involving 41 genes categorized as being involved in the zeatin biosynthesis, gibberellin biosynthesis, starch and sucrose metabolism and plant hormone signal transduction pathways. Many of the differentially expressed genes between Z571 and mZ571 related to the axillary bud type coincided with recent reports. In particular, an *miaA* gene (LOC107903664) and one *IPT* gene (LOC107912422) that were upregulated in apple [18, 47] were also found to be upregulated in mZ571. In fact, recent studies have explored the functions of *IPT* and *CKX* genes in various plant species. A study on *Pisum sativum* showed that the expression of the *PsIPT1* and *PsIPT2* genes, related to zeatin biosynthesis, in the stem nodule region differed before and after decapitation. The expression of *PsIPT* was maintained when the decapitated plants were maintained in buffer without IAA solution, whereas when IAA was added to the buffer, the expression of *PsIPT* was repressed again [14, 48]. Transgenic tobacco plants overexpressing the *IPT* gene showed higher *trans*-zeatin and zeatin riboside concentrations in the roots and stems [49]. In Arabidopsis, *IPT* single or multiple mutants exhibited significant reductions in branching [50]. According to the researchers, it is possible that the primary endogenous cytokinin synthesized mainly by adenylate *IPTs* plays a greater functional role in plant development and is more highly regulated [47, 51]. In contrast, most of the *CKX* genes were downregulated in HB-E, HB-G1 and HB-G2 (Fig. 5a), which indicates that *CKX* family genes are involved in the negative regulation of the growth and development of cotton axillary buds. This result is consistent with studies in *Arabidopsis* showing, for example, that the overexpression of the *CKX7* gene lead to decreased contents of cytokinin-related metabolites, such as N-glucoside cytokinins and cis-zeatin [47]. The *ARR-A* gene belongs to the cytokinin response gene family, which is involved in the negative regulation of cytokinin signal transduction [52]. A study on *yucca* mutants showed that the expression of the *ARR-A* gene was upregulated in association with reduced auxin levels [53]. Our determination of the concentration of zeatin in the axillary buds of Z571 and mZ571 confirmed the above conclusion.

Gibberellins are key hormones that regulate a series of activities during plant growth and development [54]. The function of the *GA20ox1* and *GA2ox1* genes has been studied in several species; *GA20ox1* positively regulates GA synthesis, while *GA2ox1* negatively regulates GA synthesis [54]. According to In our results, one *GA20ox* family gene (*GA20ox1*; LOC107888749) that was shown to be upregulated in *Jatropha curcas* [18] was also upregulated in HB-G1 and HB-G2. Most of the *GA2ox* genes were downregulated in all three stages in HB, especially the *GA2ox* family gene *GA2ox1* (LOC107899629), which showed the downregulation corresponding to a log2FoldChange of − 4.09 in HB-G1 (Fig. 5b). The GAs content of mZ571 was higher than that of Z571 in all three stages, and the concentration of GAs increased in the HB-G1 and HB-G2 stages. In fact, the zeatin and GA contents of the Z571 plant samples were lower than those of the mZ571 plant samples in the three stages, consistent with the gene expression pattern related to zeatin and gibberellin biosynthesis. For instance, in the zeatin biosynthesis pathway, the expression levels of most *IPT* and *CKX* genes in the low-budding phenotype plants were downregulated compared with those in the high-budding phenotype plants (Fig. 5a). The expression levels of *GA3ox* and *GA20ox* were downregulated in the low-budding phenotype plants (Fig. 5b). These dynamics of cytokinin and gibberellin play important roles during upland cotton axillary bud development [12, 15, 21, 55].

### Possible roles of other hormones in axillary bud development

In addition to cytokinin and gibberellin, genes related to the signal transduction of other hormones, including auxin, abscisic acid, ethylene, brassinosteroid, jasmonic acid and salicylic acid, were also differentially expressed in HB-E, HB-G1 and HB-G2 (Fig. 6). The auxin signal transduction gene *AUX1* was significantly upregulated in HB-E and HB-G1. Endogenous auxin (IAA) showed higher levels in mZ571 than in Z571, but the concentration of IAA decreased from HB-E to HB-G1 and remained stable in HB-G2. These auxin dynamics play a very important role in the development of axillary bud embryogenesis [56]. Previous studies have also shown a direct correlation between bud activity and auxin transport from the bud [14]. Our results regarding auxin signal transduction and the concentration of IAA were consistent with previous studies. Among abscisic acid signal transduction-related genes, the *PP2C* family was upregulated in HB-E and HB-G1; the *PYL* family was significantly downregulated in mZ571, with the exception of the abscisic acid receptors *PYL4* and *PYL9* in HB-E; and the *ABF* family except for *ABF5* was downregulated in mZ571. According to the investigation of the roles of abscisic acid in *Arabidopsis*, abscisic acid regulates bud outgrowth in response to the red light/far-red light ratio, thus extending the known hormonal pathways associated with the regulation of branching [57]. Our KEGG annotation results showed that light-related pathways such as photosynthesis were also significantly enriched (Table 2, Fig. 3b).

Ethylene mediates several abiotic stresses in plants [58]. Among genes related to ethylene signal transduction, *ETR* was downregulated in HB-G1, *EBF1/2* were upregulated in HB-E and HB-G2, one *ERF1B* (LOC107893200) gene was downregulated in HB-E and HB-G1, and one *ERF1B* (LOC107936230) gene was upregulated in HB-E and HB-G2. Brassinosteroid signal transduction-related genes, such as *BZR1/2*, *TCH4* and *BKI1* (LOC107917087), were downregulated or not significantly upregulated in mZ571, with the exception of one *BKI1* (LOC107958111) gene that was upregulated in HB-E and HB-G2. Among jasmonic acid signal transduction-related genes, *JAR1* was downregulated in mZ571, but *JAZ* was significantly upregulated in mZ571. Genes related to salicylic acid signal transduction and the regulatory proteins *NPR1* and *PR1* were upregulated in mZ571, and *NPR3* and *TGA* were downregulated in mZ571. These hormones may play different roles in axillary bud emergence and development in upland cotton, and the exact regulatory mechanisms involved will be discussed in our future work.

### The role of sugars in axillary bud development

In addition to plant hormones, sugars are important for plant growth and development because they provide energy and are a source of carbon for protein and cell wall synthesis [33]. Recent research has shown that sugars are one kind of important regulatory factor for bud growth and act as an early signal triggering bud activity [59]. According to our measurements, with cotton plant growth and development, the soluble sugar content of the axillary buds in the two types plants gradually increased, and the soluble sugar contents in HB-E, HB-G1 and HB-G2 were higher than those in LB-E, LB-G1 and LB-G2, respectively. These findings supported the traditional opinion that the bud is a site of the nutrient accumulation and storage and that plants with more buds show more nutrient accumulation [60]. A previous study also indicated that tubers are storage organs that accumulate starch during the development of potato [61]. Starch degradation results in an increase in the soluble sugar concentration; thus, starch may play an important role in the accumulation of endogenous soluble sugars in cells [62]. According to our starch and sucrose metabolism analysis results, *INV*, *GN5–6*, *PYG*, *GBE1* and most *TPS* genes were upregulated in the three stages of mZ571, and *WAXY* showed significant downregulation in HB-E and HB-G2 (Fig. 7). In fact, the upregulation of *PYG* promotes the degradation of starch to D-glucose 1-phosphate, which will be further converted to D-glucose. *TPS* catalyses the transformation of UDP-glucose into trehalose-6P, which is then converted into trehalose under in a reaction catalysed by *otsB*, and trehalose if finally converted into D-glucose. These gene expression patterns are consistent with the dynamic change in the soluble sugar concentration and suggest that sugars are an energy source or nutrient aiding in bud growth in high-budding phenotype cotton plants.

## Conclusion

**Fig. 8:**
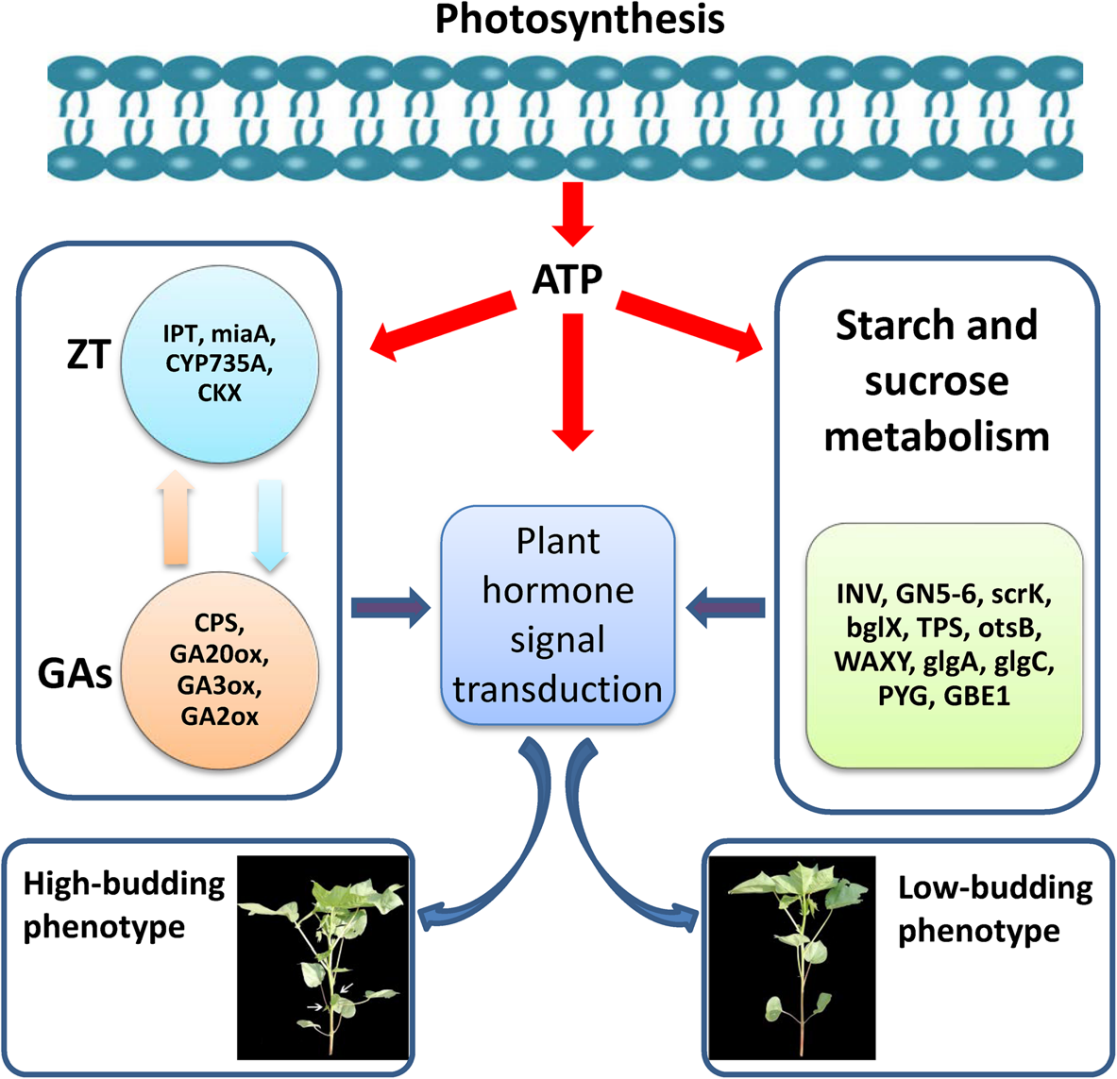
Hypothetical interaction network model of axillary bud emergence and development in upland cotton

In summary, we performed a comparative analysis of mRNA expression in the axillary buds of mZ571 and Z571 by transcriptomics. The analysis results indicated that some hormone metabolism-, hormone signal transduction- and starch metabolism-related genes showing interaction effects contribute to the divergence in axillary bud growth between mZ571 and Z571. Based on our results and previous reports, we propose a hypothetical interaction network related to axillary bud emergence and development in cotton (Fig. 8). In this model, we propose that sugars are the carbon energy source, which is essential for protein and cell wall synthesis during the emergence and development of axillary buds, hormone biosynthesis and signal transduction are the top-layer players required for cell differentiation and elongation regulating the development of axillary buds. In addition, according to KEGG analysis, we believe that photosynthesis is an important factor in the differences that arise during the development of axillary buds, which will be discussed in future work. These results further confirm the major findings previously reported for axillary buds, and important roles of genes involved in hormone biosynthesis, hormone signal transduction, and starch and sucrose metabolism were revealed. It provides us a better understanding of the complexity of the gene network involved in axillary bud emergence and development.

## Methods

### Plant materials

Upland cotton plants of two different bud types, Z571 (CCRI 9A02, low-budding phenotype) and its mZ571 mutant (high-budding phenotype), were used for this study. Z571 shows an ideal plant type for simplified cultivation and machine harvesting because of its fewer axillary buds and vegetative shoot characteristics. mZ571 shows a high-budding phenotype in the development of axillary buds compared with Z571. The plants were grown in a growth chamber at the Chinese Academy of Agricultural Sciences Cotton Research Institute (Anyang, Henan, China). The sterilized seeds were grown in a light incubator under conditions of 28 °C/25 °C and a 12/12-h light/dark cycle, 70% relative humidity and light intensity of 11,000 Lx. The seedlings were randomly divided into three groups, and each group included nine pots. The first stalk nodes of the plants were collected in the fourth week (early stage), fifth week (growth stage 1) and sixth week (growth stage 2). Three biological replicates were performed. After collection, all samples were divided into three parts: one to be used for RNA-Seq, one for endogenous hormone determination, and one for qRT-PCR. All samples were collected and stored at − 80 °C for further use.

### RNA isolation and sequencing

Total RNA was isolated from each cotton sample according to the improved CTAB extraction protocol [54]. The total RNA concentration was evaluated using a nucleic acid analyser. The construction of the cDNA library refers to the following methods. Briefly, using RNA as a template, the first strand of cDNA was synthesized with 6-base random primers and reverse transcriptase. The double-stranded cDNA library was enriched by PCR amplification and evaluated with an Agilent 2100 Bioanalyzer (Agilent Technologies, CA, USA), and the total and effective concentrations were determined by qRT-PCR. After RNA extraction, purification and library concentration, next-generation sequencing (NGS) based on the Illumina HiSeq sequencing platform was used for the paired-end (PE) sequencing of the library.

### Mapping and sequence annotation

Raw reads were pre-processed for quality filtering, in which low-quality reads, such as reads with adapters at the 3′ end and reads with an average quality score below Q20, were removed. Eventually, the clean reads were mapped to the whole genome of *Gossypium hirsutum* (GCF_000987745.1) by using the TopHat2 upgrade version HISAT2 (http://ccb.jhu.edu/software/hisat2/index.shtml) [30]. The expression levels of the unigenes were normalized by the FPKM (fragments per kilobase per million fragments) method so that the expression levels of different genes and samples could be compared.

### Identification and analysis of DEGs

DESeq was employed to analyse the differences in gene expression between samples, and only genes with an absolute log_2_ (fold change) > 1 and *p*-value < 0.05 were identified as DEGs. Gene Ontology (GO) annotation was performed by using the Blast2GO program. The gene list and the numbers of each term were calculated for the annotated DEGs. The hypergeometric distribution method was selected to calculate the *p*-values (the criterion for significant enrichment was a p-value < 0.05), and the GO terms with significantly enriched DEGs were identified. Kyoto Encyclopedia of Gene and Genomes (KEGG) annotations were obtained from the KEGG automatic annotation server [63].

### Determination of endogenous hormones and soluble sugars

According to previous studies, ZT is an important cytokinin used to promote plant regeneration [64, 65]. ZT induces the formation of a greater number of shoot primordia, many of which are converted into individual shoots in upland cotton tissue culture [31]. IAA and GAs are commonly applied in plant growth regulation research [21, 33]. Therefore, the concentrations of ZT, IAA and GAs in axillary buds were tested using high-performance liquid chromatography (HPLC), and the soluble sugar contents were determined using a colorimetric method. Three biological repeats were performed.

### Validation of RNA-Seq by qRT-PCR

Here, to verify the validity of the transcriptome data, sixteen genes were chosen for qRT-PCR. The specific primers were designed by using Primer Premier 5 software (Table S1), and a Fast Quant RT Kit (Tiangen Biotech) was used to conduct reverse transcription. qRT-PCR was performed in an IQ5 fluorescent quantitative PCR system (Bio-Rad, USA) by using an Ultra SYBR Mixture (with ROX 1) kit (CWBIO, Beijing, China). The results were analysed by the 2^−ΔΔCT^ method with normalization against the *UBQ7* gene [54]. Each amplification reaction was performed in a total volume of 10 μL, containing 5 μL of 2× Ultra SYBR Mixture, 0.4 μL of each gene-specific primer (10 μmol/L), 0.7 μL of diluted cDNA, and 3.5 μL of sterile distilled water. The program for each reaction was 95 °C for 10 min, followed by 40 cycles of 95 °C for 20 s and 65 °C for 30 s. At the end of the reaction, the specificity of the amplified product was determined by the melting curve method. Three biological and technical replicates were performed to ensure reliability.

## Supplementary information

**Additional file 1: Figure S1**. The eggNOG classification of assembled unigenes. X-axis: the name of 26 categories in eggNOG, Y-axis: the number of unigene annotated in the category.

**Additional file 2: Table S1.** Primer sequences of genes used for quantitative RT-PCR verification.

**Additional file 3: Table S2.** Summary of raw data analysis.

**Additional file 4: Table S3.** Summary of RNA-Seq Map.

## Data Availability

The RNA-Seq data of all samples have been submitted to GenBank of the National Center for Biotechnology Information (NCBI: http://www.ncbi.nlm.nih.gov/sra), and the Sequence Read Archive (SRA) accession number is PRJNA598982.

## Abbreviations

PAT: Polar auxin transport
IPT: Adenosine phosphate-isopentenyl transferase
TCP: TEOSINTE BRANCHED1, CYCLOIDEA, PCF
*BRC1*: *BRANCHED1*
*FC1*: Fine culm 1
*RBOH1*: Respiratory burst oxidase homolog 1
GO: Gene ontology
*WFI1*: Whitefly induced 1
*PIN*: *PIN-FORMED*
qRT-PCR: Quantitative real-time PCR
NGS: Next-Generation Sequencing
DEGs: Differentially expressed genes
ZT: Zeatin
GAs: Gibberellins
IAA: Indole acetic acid
HPLC: High-performance liquid chromatography
*CKX*: Cytokinin dehydrogenase
*GN5–6*: Glucan endo-1,3-beta-glucosidase 5/6
*TPS*: Trehalose 6-phosphate synthase/phosphatase
*WAXY*: Granule-bound starch synthase
