# Supplementary figures and images for: Transcriptome analyses provide insights into the homeostatic regulation of axillary buds in upland cotton (G. hirsutum L.)
Source: BMC Plant Biol. 2020 May 24;20:228. doi: 10.1186/s12870-020-02436-x (PMC7245931; doi:10.1186/s12870-020-02436-x)

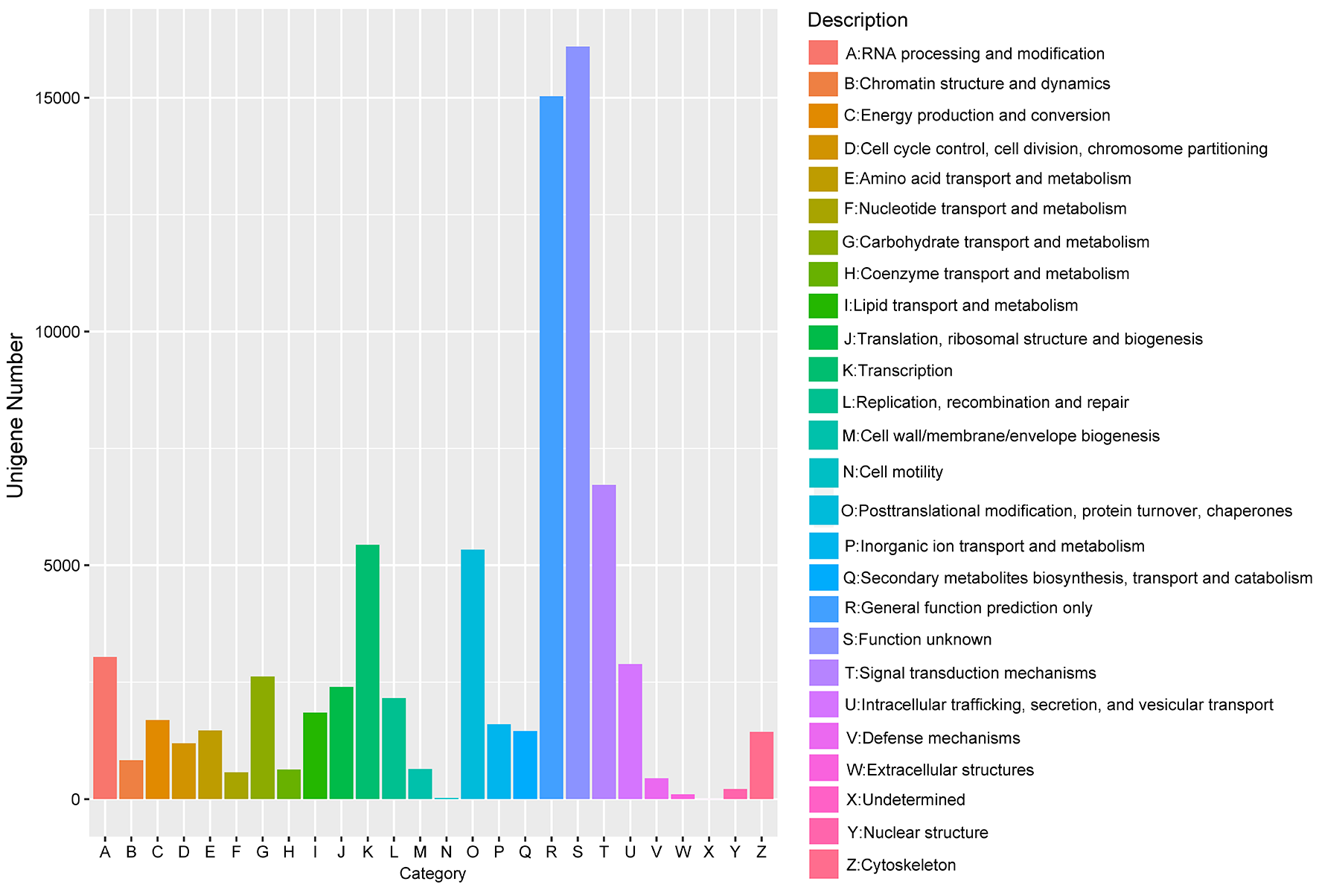


**Fig. S1**

Supplement: Supplementary file 1 — Additional file 1: Figure S1. The eggNOG classification of assembled unigenes. X-axis: the name of 26 categories in eggNOG, Y-axis: the number of unigene annotated in the category. [file 12870_2020_2436_MOESM1_ESM.docx]
