# Supplementary material for: Transcriptome analyses provide insights into the homeostatic regulation of axillary buds in upland cotton (G. hirsutum L.)
Source: BMC Plant Biol. 2020 May 24;20:228. doi: 10.1186/s12870-020-02436-x (PMC7245931; doi:10.1186/s12870-020-02436-x)
Supplement: Supplementary file 2 — Additional file 2: Table S1. Primer sequences of genes used for quantitative RT-PCR verification. [file 12870_2020_2436_MOESM2_ESM.docx]

| **Table S1** Primer sequences of genes used for quantitative RT-PCR verification | | | |
| --- | --- | --- | --- |
| **Gene name** | **Gene symbol** | **Forward Primer (5’→3’)** | **Reverse Primer (5’→3’)** |
| *UBQ7* | DQ116441.1 | AAGAAGAAGACCTACACCAAGCC | GCCCACACTTACCGCAATA |
| *CYP735A* | LOC107945836 | GCTTAAGAGTTATTGCGGCCA | TCAGCAGTAAGCCGAGACAT |
| *CKX* | LOC107932015 | CCTGGGAAGCTTTTGAAGACC | TGGCCTGGTGACAACAACAT |
| *CPS* | LOC107890714 | CGGAAATGGGTCTGGCTGAT | CCGCTCTTGCGACCTTTCAA |
| *GA3ox* | LOC107937357 | TGAGCATCGGCCTTTAGCTT | TGGAATTACAAGTGCCGGGG |
| *CYP734A* | LOC107894248 | AACAAAGAAGCTGCCCAAATGTCC | TCCCGTATTTGTTGATCCAGGAG |
| *CN5-6* | LOC107931579 | TCCCTCCCCAAGAGATGGAAA | GAGGGCTTTGGCTCTAATTCAT |
| *WAXY* | LOC107930241 | TTGGTGATGTTCTTGGCGGA | GTGACACAAGTGTCCCATGC |
| *AUX/IAA* | LOC107927655 | TGGGAGATGTTTGTTGACTCAT | GAGCGAGCCCTATTGCCTC |
| *SAUR* | LOC107949392 | ATGGTCTCCCTGTCGACGTA | ACTCGGGGTGTGTCAAGAAC |
| *ARR-A* | LOC107890909 | TTATGGAGGTTGCTGGTGAGG | CTCTTGCCCCACTCTCCAC |
| *TF* | LOC107888469 | CACCTCAAAGTCCCACGGTA | TCCCATTTTCGGTCTGCGAT |
| *PP2C* | LOC107906580 | AGCACTACGTCACGGTACAA | GCTCTACAGAGGGAGGCATT |
| *TCH4* | LOC107919247 | TCGTTGCTTCTCTCTTTTATTCAGC | TGCCATCACCCCAAGTAACA |
| *PsbS* | LOC107905013 | TGCTGCTATAAACCCTGGGAC | AAAGCCCAGTTAAGAAACTTCAG |
| *PsaD* | LOC107951786 | ACTTCAAAGTCCCTGGCTCC | TGCAGCCCTGATGCTAGTTC |
| *ATPF1D* | LOC107948155 | AACACCGACGTCCCAACAAT | GAGCTGCCTTAGATCAACCGT |
