# Supplementary material for: Transcriptome analyses provide insights into the homeostatic regulation of axillary buds in upland cotton (G. hirsutum L.)
Source: BMC Plant Biol. 2020 May 24;20:228. doi: 10.1186/s12870-020-02436-x (PMC7245931; doi:10.1186/s12870-020-02436-x)
Supplement: Supplementary file 3 — Additional file 3: Table S2. Summary of raw data analysis. [file 12870_2020_2436_MOESM3_ESM.docx]

| **Table S2** Summary of raw data analysis | | | | | | |
| --- | --- | --- | --- | --- | --- | --- |
| **Sample** | **Reads No.** | **Bases (bp)** | **Q30 (bp)** | **N (%)** | **Q20 (%)** | **Q30 (%)** |
| HB-E-1 | 47502956 | 7172946356 | 6786477440 | 0.002132 | 97.72 | 94.61 |
| HB-E-2 | 50208022 | 7581411322 | 7160572795 | 0.001999 | 97.66 | 94.44 |
| HB-E-3 | 44107372 | 6660213172 | 6324444080 | 0.001869 | 97.94 | 94.95 |
| HB-G1-1 | 50119320 | 7568017320 | 7167859976 | 0.002271 | 97.80 | 94.71 |
| HB-G1-2 | 48370668 | 7303970868 | 6938871147 | 0.002519 | 97.94 | 95.00 |
| HB-G1-3 | 43007084 | 6494069684 | 6168029773 | 0.001951 | 97.95 | 94.97 |
| HB-G2-1 | 46911274 | 7083602374 | 6746129875 | 0.001998 | 98.09 | 95.23 |
| HB-G2-2 | 45027192 | 6799105992 | 6457513245 | 0.002199 | 97.95 | 94.97 |
| HB-G2-3 | 44946016 | 6786848416 | 6450302386 | 0.001904 | 97.99 | 95.04 |
| LB-E-1 | 48545680 | 7330397680 | 6974957421 | 0.001829 | 98.04 | 95.15 |
| LB-E-2 | 41286088 | 6234199288 | 5902133509 | 0.001913 | 97.80 | 94.67 |
| LB-E-3 | 42343962 | 6393938262 | 6067472218 | 0.001902 | 97.91 | 94.89 |
| LB-G1-1 | 41151172 | 6213826972 | 5910894459 | 0.001762 | 98.01 | 95.12 |
| LB-G1-2 | 52071024 | 7862724624 | 7471986213 | 0.001974 | 97.97 | 95.03 |
| LB-G1-3 | 53978602 | 8150768902 | 7758220327 | 0.002045 | 98.05 | 95.18 |
| LB-G2-1 | 50839398 | 7676749098 | 7322332624 | 0.002295 | 98.14 | 95.38 |
| LB-G2-2 | 49547746 | 7481709646 | 7104902430 | 0.002088 | 97.95 | 94.96 |
| LB-G2-3 | 45097982 | 6809795282 | 6475196432 | 0.002228 | 98.00 | 95.08 |

"HB-E" and "LB-G" for "high-budding phenotype, early stage" and "low-budding, growth stage", respectively.
