# Supplementary material for: Transcriptome analyses provide insights into the homeostatic regulation of axillary buds in upland cotton (G. hirsutum L.)
Source: BMC Plant Biol. 2020 May 24;20:228. doi: 10.1186/s12870-020-02436-x (PMC7245931; doi:10.1186/s12870-020-02436-x)
Supplement: Supplementary file 4 — Additional file 4: Table S3. Summary of RNA-Seq Map. [file 12870_2020_2436_MOESM4_ESM.docx]

| **Table S3** Summary of RNA-Seq Map | | | | |
| --- | --- | --- | --- | --- |
| **Sample** | **Clean Reads** | **Total Mapped** | **Multiple Mapped** | **Uniquely Mapped** |
| HB-E-1 | 44329246 | 41748694 (94.18%) | 4331669 (10.38%) | 37417025 (89.62%) |
| HB-E-2 | 46935956 | 44574610 (94.97%) | 4603146 (10.33%) | 39971464 (89.67%) |
| HB-E-3 | 41303400 | 39314348 (95.18%) | 4039373 (10.27%) | 35274975 (89.73%) |
| HB-G1-1 | 46865742 | 44480234 (94.91%) | 4553772 (10.24%) | 39926462 (89.76%) |
| HB-G1-2 | 45240268 | 42859866 (94.74%) | 4482898 (10.46%) | 38376968 (89.54%) |
| HB-G1-3 | 40269048 | 38267694 (95.03%) | 3868379 (10.11%) | 34399315 (89.89%) |
| HB-G2-1 | 43922580 | 41509718 (94.51%) | 4208187 (10.14%) | 37301531 (89.86%) |
| HB-G2-2 | 42169504 | 39693454 (94.13%) | 4080642 (10.28%) | 35612812 (89.72%) |
| HB-G2-3 | 42062384 | 40083702 (95.30%) | 3871141 (9.66%) | 36212561 (90.34%) |
| LB-E-1 | 45431676 | 43370102 (95.46%) | 4469034 (10.30%) | 38901068 (89.70%) |
| LB-E-2 | 38701060 | 36786020 (95.05%) | 3873808 (10.53%) | 32912212 (89.47%) |
| LB-E-3 | 39697058 | 37785918 (95.19%) | 3944819 (10.44%) | 33841099 (89.56%) |
| LB-G1-1 | 38396184 | 36570366 (95.24%) | 3781494 (10.34%) | 32788872 (89.66%) |
| LB-G1-2 | 48605072 | 46096724 (94.84%) | 4968248 (10.78%) | 41128476 (89.22%) |
| LB-G1-3 | 50414606 | 47858792 (94.93%) | 5051149 (10.55%) | 42807643 (89.45%) |
| LB-G2-1 | 47460734 | 44829608 (94.46%) | 4715725 (10.52%) | 40113883 (89.48%) |
| LB-G2-2 | 46382786 | 43591916 (93.98%) | 4768041 (10.94%) | 38823875 (89.06%) |
| LB-G2-3 | 42123918 | 39627908 (94.07%) | 4303799 (10.86%) | 35324109 (89.14%) |

"HB-E" and "LB-G" for "high-budding phenotype, early stage" and "low-budding, growth stage", respectively.
